# The role of GLP-1 in the pathophysiology and treatment of sepsis: a narrative review

**DOI:** 10.3389/fmed.2025.1612034

**Published:** 2025-11-21

**Authors:** Nemanja Dimic, Marko Djuric, Maja Vejapi, Irina Nenadic, Marina Bobos, Suzana Bojic, Predrag Savic, Miljan Milanovic, Predrag Stevanovic

**Affiliations:** University Hospital Center Dr Dragiša Mišović, Belgrade, Serbia

**Keywords:** GLP-1 receptor agonists, Sepsis, muscle atrophy, inflammation, oxidative stress

## Abstract

**Background:**

Sepsis remains a leading cause of morbidity and mortality in critically ill patients, often complicated by sepsis-induced myopathy (SIM), systemic inflammation, and multiorgan dysfunction. Glucagon-like peptide-1 receptor agonists (GLP-1RAs), initially developed for the treatment of type 2 diabetes, have demonstrated pleiotropic effects that may be beneficial in the septic context.

**Objective:**

This review aims to explore the significance of GLP-1 receptors in the sepsis mechanism, as well as the therapeutic potential of GLP-1RAs in sepsis treatment, with a particular emphasis on their role in modulating inflammation, improving metabolic and endothelial function, and mitigating systemic inflammatory response syndrome (SIRS).

**Methods:**

A comprehensive synthesis of preclinical and clinical studies was conducted, focusing on the cellular mechanisms and systemic outcomes of GLP-1RA therapy in various models of sepsis and critical illness.

**Results:**

GLP-1RAs attenuate inflammation by suppressing NF-κB and p38 MAPK pathways, reduce oxidative stress, enhance insulin sensitivity, and promote mitochondrial and endothelial stability. In skeletal muscle, they downregulate atrophy-associated genes (MuRF1, MAFbx) and upregulate myogenic factors (MyoD, MyoG), thereby improving perfusion and energy metabolism. Central GLP-1R signaling plays a crucial role in neuroimmune modulation and organ protection. Notably, these agents also increase adiponectin levels, which may further contribute to vascular integrity and anti-inflammatory effects during sepsis.

**Conclusion:**

GLP-1RAs represent a novel and multifaceted therapeutic strategy for sepsis and its complications. They show promise as adjunctive therapy in sepsis due to their anti-inflammatory, antioxidant, and endothelial-protective actions. Experimental and limited clinical data suggest improved organ function and survival, but further human studies are needed to confirm efficacy, safety, and optimal treatment strategies.

## 1 Introduction

According to the latest iteration of the definition, sepsis is a life-threatening organ dysfunction resulting from a dysregulated host response to infection (1). Low- and middle-income countries (LMICs) carry approximately 85% of the global burden, with age-standardized incidence rates often exceeding 1,500 cases per 100,000 population and mortality rates surpassing 40%. The economic impact in these settings is profound, with average treatment costs around $10,000 per patient-often exceeding the monthly income of a typical household. In contrast, high-income countries (HICs) report lower age-standardized incidence rates, typically ranging from 400 to 1,000 per 100,000. Mortality in HICs has declined significantly over the past two decades, with current estimates between 15–20%, yet the economic burden remains substantial, with per-patient costs ranging from $20,000 to $50,000 (2, 3).

### 1.1 Overview of sepsis pathophysiology

The immunological response to the entry of microorganisms into the body and the resulting infection involves the transmigration of leukocytes and the release of cytokines, prostaglandins and other inflammatory molecules (4). Such a response is how the body fights against bacteria, viruses, fungi or parasites. The infection becomes sepsis when that response becomes excessive, harmful, disbalanced and leads to systemic inflammation, probably due to immunosuppression (5). Innate immune system as a first line of defense includes macrophages, natural killer (NK) cells, phagocytes and neutrophils (6). Neutrophil migration to the site of the infection is the first event that happens in the first few hours after pathogen entry. It is expected that this event eliminates pathogens and promotes healing (7). Cytokines, chemokines and prostaglandins can help control inflammatory response, but on the other hand, if their release is dysregulated, it can worsen a patient’s condition. Complex immune response activates different mechanisms during the development of sepsis, both proinflammatory and anti-inflammatory, and as a result, immunosuppression occurs quickly. Consequent immunosuppression causes a disbalance of inflammatory mediators (8). During the infection, the host’s organism fights against it with its defense mechanisms, unlike sepsis, when the host’s response to the infection is inadequate (9). Tumor necrosis factor-*α* (TNF-α) is a major mediator of sepsis. Its formation, by macrophages and neutrophils, is stimulated by bacteria and viruses. It initiates the inflammatory response by binding to its receptors, TNF-*α* type 1 receptor expressed on all human tissues and type 2 receptor mainly expressed in immune cells (10). The primary proinflammatory mediators like TNF-*α*, Interleukin-1 (IL-1) and Interleukin-6 (IL-6) are produced when pathogens’ antigens bind to toll-like receptor-4 (TLR-4). The importance of TNF-*α* can be explained by the fact that mutation of TLR-4 affects patients’ development of sepsis and its degree due to different responsiveness of lipopolysaccharide (LPS) (11). TNF-*α* exerts its effects by activating the intracellular signaling pathway NF-κB, further stimulating the multi-protein complex, NOD-like receptor protein 3 (NLRP3) inflammasome (12). Besides NF-κB, NADPH oxidase can also be activated by TNF-*α* in the endothelial and vascular smooth muscle cells (13). As a result, reactive oxygen species (ROS) production is induced, and nitric oxide (NO) levels are decreased (14). A disbalance between excessive production of ROS and the body’s ability to overcome it causes oxidative stress (15). Mitochondrial dysfunction also contributes to sepsis-induced myopathy (SIM). The ubiquitin-proteasome system, autophagy, and calpains are the three major pathways associated with protein degradation, and they still appear to be a central component of SIM. ROS is considered the crucial component of the activation of the ubiquitin-proteasome system. It has also been believed that IL-6 induces skeletal muscle wasting. Yang B et al. demonstrated that IL-6 deficiency attenuated the development of SIM through upregulation of the peroxisome proliferator-activated *γ* coactivator-1α and inhibition of mitochondrial ROS production (16). The inability of the organism to defend itself and the vigorous activity of proinflammatory mediators lead to hypoperfusion and ischemia in many organs (17). The pathogenesis of sepsis is a complex cascade of numerous mediators that cause cellular dysfunction, coagulopathy, endothelial dysfunction and changes in the cardiovascular system. Consequent endothelium damage results in increased permeability of the endothelium due to disruption of cell–cell junction (18). Hyperpermeability of vascular endothelium enables invasion of macrophages and neutrophils into the vessels and release of thromboplastin from the endothelium of blood vessels, which is responsible for the activation of coagulation (19). Sepsis-induced coagulopathy is characterized by the formation of microvascular thrombi that can result in inadequate organ perfusion (20). The resulting organ dysfunction leads to further spread of the inflammatory process, which can develop into Multiorgan Dysfunction Syndrome (MODS), due to a disbalance between protective and harmful mechanisms. The body is then minimally capable of regenerating damaged tissue (21).

Glucagon-like peptide-1 receptor (GLP-1R) is widely expressed G protein-coupled receptor, mediates the effects of Glucagon-like peptide-1 (GLP-1), a peptide derived from proglucagon and produced in intestinal L-cells, pancreatic *α*-cells, and specific brain regions (22). By enhancing insulin secretion, inhibiting glucagon release, delaying gastric emptying, and suppressing appetite, GLP-1R agonists (GLP-1RAs) have demonstrated efficacy in managing type 2 diabetes and obesity (23). More recently, their anti-inflammatory, cardioprotective, and neuroprotective properties have been explored in conditions such as cardiovascular disease (24), non-alcoholic fatty liver disease (25), osteoarthritis (26), rheumatoid arthritis (27), Alzheimer’s (28), and Parkinson’s disease (29). Clinical data support their ability to reduce risks of heart failure (30), atherosclerosis (31), and hypertension (32), highlighting their potential as systemic therapeutics. The development of stable, long-acting GLP-1RAs marks significant advancement, positioning these agents at the forefront of translational research across multiple disease domains.

Despite the rapid expansion of GLP-1RAs applications in fields such as endocrinology, cardiology, and neurology, their potential relevance in sepsis remains relatively underrepresented in the literature. This narrative review aims to synthesize current evidence on the physiological and therapeutic relevance of GLP-1 signaling in sepsis, highlight mechanistic insights from preclinical studies, and assess the translational potential of GLP-1RAs in modulating systemic inflammation, metabolic dysregulation, and organ dysfunction in septic states.

## 2 Methods

A comprehensive literature search was performed using PubMed, Scopus, and Web of Science databases from their inception through June 2024. The search strategy combines Medical Subject Headings (MeSH) and keywords including “GLP-1,” “glucagon-like peptide-1 receptor,” “GLP-1 receptor agonist,”” sepsis,”” systemic inflammation,” “infection,”” immune response,” “critical illness,”” MODS,” “multiple organ dysfunction,” “cytokines” and “sepsis treatment.” Articles were screened for relevance based on titles and abstracts, with full-text evaluation of studies addressing the mechanistic or therapeutic implications of GLP-1 signaling in experimental or clinical models of sepsis. Reference lists of included articles and relevant reviews were also manually screened to identify additional sources. Eligible studies were limited to English-language, peer-reviewed publications. As this is a narrative rather than a systemic review, no formal quality assessment or meta-analysis was performed. The synthesis of findings was organized thematically according to mechanistic pathways and clinical relevance.

## 3 Results

**Figure 1:**
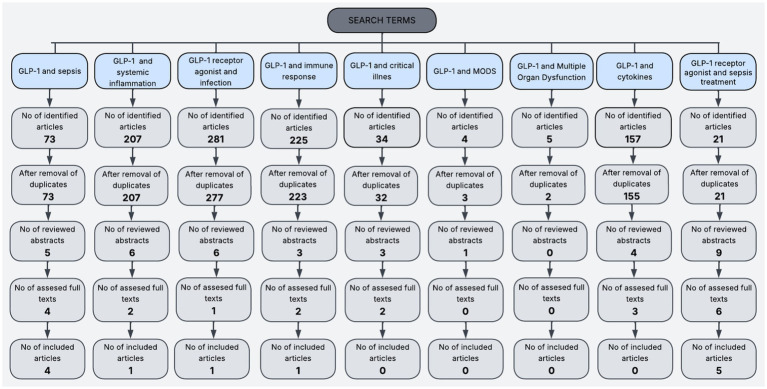
Flowchart of steps for studies selection for each search term.

Using predefined search terms such as “*GLP-1”* and “*sepsis,”* we initially identified a total of 73 articles through electronic database searches (e.g., PubMed, Scopus, Web of Science). After removing duplicates, five abstracts were screened for relevance based on the predefined inclusion and exclusion criteria. Following abstract screening, four full-text articles were assessed for eligibility. Ultimately, four studies were included. Using the search terms “*GLP-1”* and *“systemic inflammation,”* 207 articles were identified. After removing duplicates, six abstracts were screened based on relevance, and two full texts were assessed. Ultimately, one study was included. Using the keywords “*GLP-1 receptor agonist”* and *“infection,”* a total of 281 articles were identified. After removing four duplicates, a total of 277 articles remained. Based on relevance, six abstracts were reviewed and one ull text. Ultimately, 1 article was included in this narrative review. Using the search terms *“GLP-1”* and “*immune response,”* 225 articles were identified. After removing two duplicates, a total of 223 articles were identified. Based on relevance, three abstracts were screened, and two full texts were assessed. Ultimately, one study was included. Using the search terms *“GLP-1”* and *“critical illness,”* a total of 34 articles were identified, after removing two duplicates. Following this, 32 articles were selected, and three abstracts were reviewed. Following abstract screening, two full texts were assessed, and no articles were included in this narrative review. Using a search terms *“GLP-1”* and “*MODS,”* four articles were identified. After removing duplicates, a total of three articles remained. Following the screening of one abstract, no full texts were reviewed (access was restricted due to payment requirements), and no articles were included in this narrative review. Using the keyword “*GLP-1*” and “*multiple organ dysfunction*,” five articles were identified. After removing three duplicates, a total of two articles were screened. Based on the predefined relevance criteria, none of the abstracts met the inclusion criteria. Consequently, no full texts were assessed, and no studies were included. Using keywords such as “*GLP-1*” and “*cytokines*,” a total of 157 articles were identified. After removing two duplicates, a total of 155 articles remained. Based on relevance criteria, four abstracts were reviewed, and three full texts were selected. Finally, none of the articles were included in this narrative review. Using keywords such as “*GLP-1 receptor agonist*” and “*sepsis treatment*,” a total of 21 articles were identified, with no duplicates. Based on relevance, nine abstracts were reviewed, and six full texts were selected. Ultimately, five articles were included in this narrative review. Figure 1 illustrates results for each search term detailing the number of studies included and the selection process.

## 4 Overview of the GLP-1 mechanism

GLP-1 is an incretin hormone composed of 30–31 amino acids, produced by post-translational processing of proglucagon and secreted by intestinal L-cells in response to nutritional and inflammatory stimuli, as well as by neurons in the brainstem. GLP-1 acts through the G protein-coupled receptor, which is expressed in the pancreas, lungs, heart, nervous system, and gastrointestinal tract. Activation of GLP-1R leads to an increase in intracellular cAMP and/or calcium, triggering signaling pathways including protein kinase A (PKA), exchange proteins directly activated by cAMP (Epac-2), phospholipase C (PLC), and extracellular signal-regulated kinase 1 and 2 (ERK1/2), thereby mediating effects on glycemic regulation, immune process, and inflammation. Its actions include stimulation of glucose-dependent insulin secretion, inhibition of glucagon, preservation of pancreatic *β*-cells, and anti-inflammatory effects in various disease models. However, under physiological conditions, GLP-1 is rapidly degraded by the enzyme dipeptidyl peptidase-4 (DPP-4) (33).

### 4.1 Mechanism of anti-inflammatory effects

**Figure 2:**
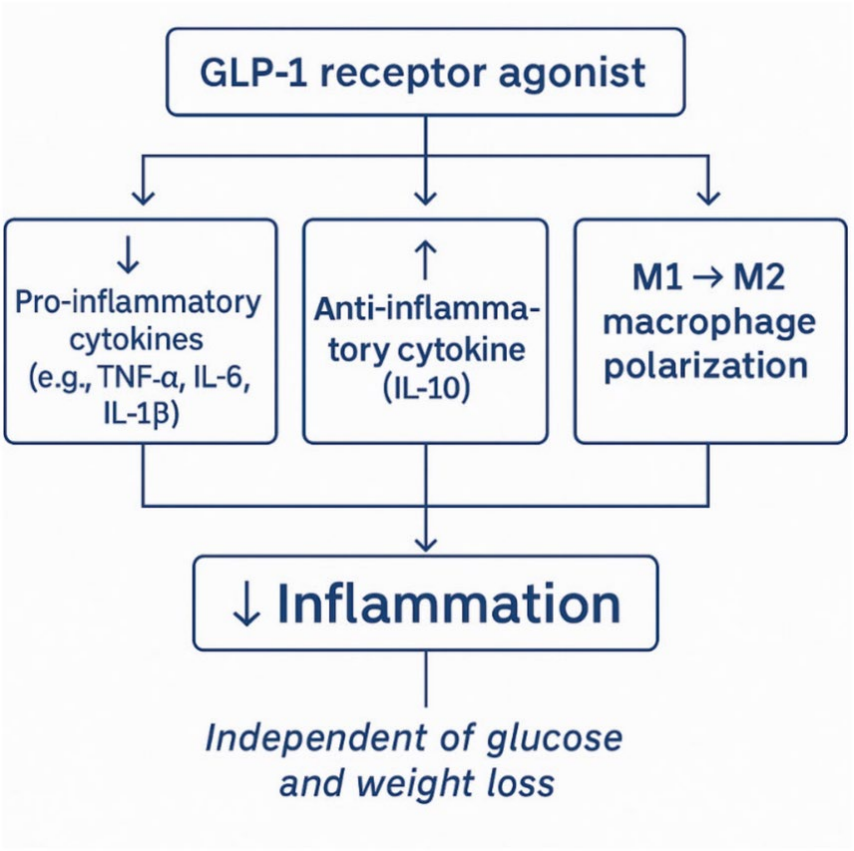
Anti-inflammatory effects of GLP-1 agonists.

**Figure 3:**
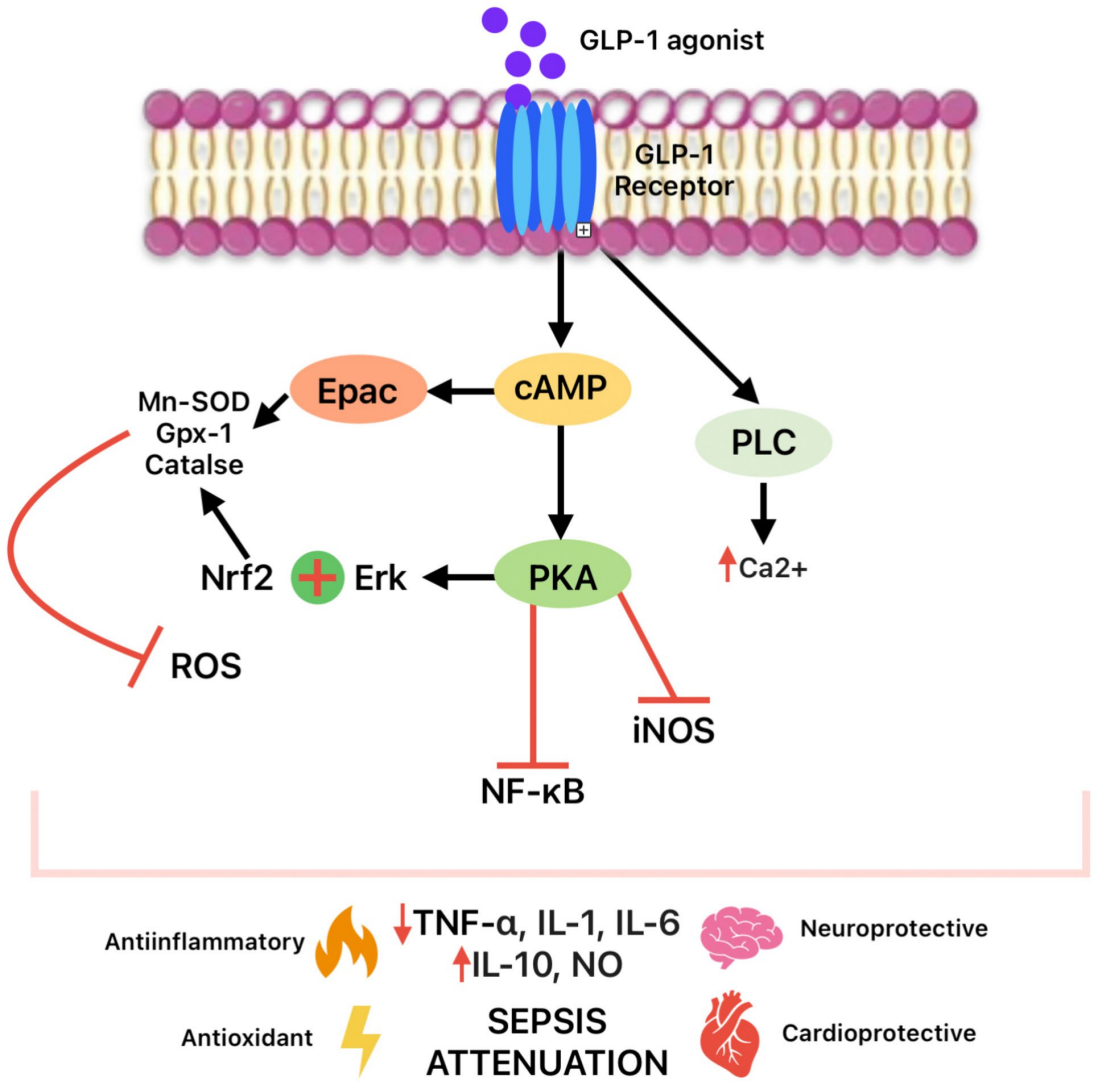
GLP-1 RA signaling pathways in sepsis attenuation.

GLP-1 exhibits potent anti-inflammatory effects. It has been demonstrated that GLP-1Rs are expressed on various immune cells, including T and B lymphocytes, monocytes, macrophages, neutrophils, eosinophils, and CD34 + progenitor cells. They act both directly, through receptor activation on immune cells, and indirectly, through glycemic control and weight reduction. (34) Experimental and clinical studies demonstrate that GLP-1RAs reduce the production of proinflammatory cytokines (e.g., TNF- *α*, IL-6, Interleukin-1β - IL-1β) and chemokines (e.g., Monocyte Chemoattractant Protein-1 - MCP-1, CXCL10), while increasing anti-inflammatory cytokines such as Interleukin-10 (IL-10). They also promote macrophage polarization from the proinflammatory M1 phenotype to the anti-inflammatory M2 phenotype (Figure 2). These effects are observed independently of glucose levels and weight loss (35). The mechanism involves inhibition of the NF-κB signaling pathways, a central regulator of genes involved in inflammation (Figure 3). Thus, GLP-1 may mitigate the cytokine storm characteristic of the early phase of sepsis and contribute to preventing multiple organ failure. Studies have shown that GLP-1RA administration decreases serum levels of inflammatory cytokines, improves hemodynamic parameters, and reduces mortality (36). Liraglutide (Lira), a GLP-1 analog, may have anti-inflammatory properties. Some of the anti-inflammatory mechanisms of this molecule in sepsis include reducing IL-6 and inducible nitric oxide synthase (iNOS). Additionally, liraglutide can also normalize endothelial nitric oxide synthase (eNOS) expression, which may contribute to its anti-inflammatory effects by increasing NO bioavailability and protecting the endothelium from inflammation-induced damage (37). Sazgarnejad and colleagues conducted a literature review using PubMed in April 2021 with keywords related to GLP-1, COVID-19, and inflammation. After screening abstracts and full texts, they concluded that GLP-1RA exhibit anti-inflammatory and anti-apoptotic effects across multiple organ systems, including cardiovascular, respiratory, and endocrine systems. These effects are primarily mediated through the suppression of pro-inflammatory cytokines (38).

## 5 Effects of GLP-1 receptor agonists on biomarkers of inflammation

In a systematic review and meta-analysis conducted by Bray et al., data from 40 randomized controlled trials were analyzed to examine the effects of GLP-1RAs on biomarkers of inflammation and oxidative stress in patients with type 2 diabetes. Although the study primarily reports clinical outcomes, changes in specific biomarkers indirectly suggest potential mechanisms of action of GLP-1RAs. A significant reduction in pro-inflammatory molecules, including C-reactive protein (CRP), TNF-*α*, and IL-6, was observed, indicating the inhibition of inflammatory pathways, likely at the level of immune system cells and adipocytes signaling. Concurrently, an increase in adiponectin, an anti-inflammatory adipokine, was observed, suggesting a reprogramming of adipocyte secretion toward a protective phenotype. In terms of oxidative stress, levels of malondialdehyde (MDA), 8-iso-prostaglandin F2α, and 8-hydroxy-2’deoxyguanosine (8-OHdG) were significantly reduced, implying that GLP-1RAs reduce lipid peroxidation and oxidative DNA damage. Although molecular mechanisms are not explicitly described, the authors highlight that these biomarkers are reliable indicators of the anti-inflammatory and antioxidant potential of GLP-1RAs, which may contribute to their cardioprotective and renoprotective actions in inflammatory conditions such as sepsis (35).

## 6 GLP-1 role in sepsis pathophysiology

In addition to its significant role in regulating glycemia, GLP-1 exhibits numerous pleiotropic properties that may be relevant in the pathophysiology of sepsis. Sepsis is a severe condition characterized by systemic inflammation, metabolic disturbances, oxidative stress, endothelial dysfunction, and ultimately, poor tissue perfusion. GLP-1 may act protectively on all these processes through various related mechanisms. It is well known that sepsis often leads to hyperglycemia and insulin resistance, which can worsen disease outcome. Recent evidence highlights GLP-1RAs as a promising adjunctive therapy in critical conditions due to their protective effects: they improve endothelial function, reduce ischemia–reperfusion injury, and regulate autonomic tone in the cardiovascular system; in the central nervous system, they exert neuroprotective effects by reducing neuroinflammation and pyroptosis; in the lungs, they alleviate acute respiratory distress syndrome (ARDS) by reducing cytokine production, stimulating surfactant secretion, and preserving alveolar-capillary barrier; in the kidneys, they reduce acute injury and preserve filtration function; in the gastrointestinal tract, they module the microbiota, strengthen the intestinal barrier, and reduce systemic inflammation through the gut-lung axis; additionally, the mitigate liver inflammation, support pancreatic *β*-cell survival, and improve insulin sensitivity and metabolic stability in intensive care units (ICUs) (39). Pegylated forms of Exedin-4 can significantly reduce organ damage caused by sepsis (cecal ligation and puncture model) by decreasing vascular permeability and inhibiting the interaction between leukocytes and endothelial cells (40). According to a study by Yang et al., GLP-1RAs improve glycemic control and insulin sensitivity in septic patients, thereby alleviating metabolic stress and reducing the risk of organ damage. As previously mentioned, they stabilize mitochondrial function and reduce oxidative stress, and inflammation by inhibiting signaling pathways, such as NF- κB, thereby contributing to cellular homeostasis during sepsis (41). Shah and colleagues analyzed ten small clinical studies (with a maximum of 40 participants each), five of which included patients with sepsis. Participants received GLP-1 infusions, which resulted in a reduction in blood glucose levels, decreased glycemic variability over time, and enhanced endogenous insulin secretion. The authors suggest that therapies aimed at enhancing incretin activity may offer similar benefits in the management of critically ill patients with sepsis, primarily by supporting euglycemia and modulating the host’s inflammatory response (42).

### 6.1 Antioxidant effects

GLP-1 receptor agonists exhibit potent antioxidant effects in sepsis by enhancing the expression and activity of key enzymes, such as superoxide dismutase (SOD), glutathione peroxidase (GPx), and catalase, which effectively neutralize ROS that are major mediators of cellular injury during sepsis. Binding of GLP-1 to the Gα_s-coupled receptor increases intracellular cAMP, which activates PKA, and the Epac pathway; the Epac-dependent signal induces explicitly expression of Mn-SOD, GPx-1, and catalase in cardiomyocytes and hepatocytes, reducing ROS and protecting cells from oxidative stress (43). Concurrently, GLP-1 stimulates the translocation of the transcription factor nuclear factor erythroid 2-related factor 2 (Nrf2) into the nucleus via the PKA/ERK signaling pathway, where Nrf2 induces transcription of genes for SOD, GPx, catalase, thioredoxin, and other antioxidant enzymes, further enhancing the cellular antioxidant defense system. The role of semaglutide (an oral GLP-1 analog) in defending against oxidative stress is well-known. This drug can activate Nrf2. It drives the expression of genes responsible for producing antioxidant enzymes, and it can also increase the activity of antioxidant enzymes like SOD and catalase, which play a crucial role in neutralizing free radicals. Additionally, semaglutide has been shown in experimental models to increase GPx activities and decrease lipid peroxidation (MDA levels) through the PI3K/Akt/Nrf2 signaling pathway (44). As a result of these mechanisms, GLP-1 agonists stabilize mitochondrial function, prevent oxidative damage to lipids, proteins, and DNA, and preserve the integrity of cellular structures in vital organs, including the heart, kidneys, endothelium, and immune cells, which is crucial for preventing organ complications in sepsis.

### 6.2 GLP-1 role in metabolic stability

GLP-1 plays a key role in maintaining metabolic stability, particularly in critical conditions such as sepsis, where the organism often enters a catabolic state with disturbed glycemic regulation. Unlike many hypoglycemic drugs, GLP-1 regulates glucose levels in a glucose-dependent manner, significantly reducing the risk of hypoglycemia. The mechanism involves enhanced insulin secretion from pancreatic *β*-cells only when glucose levels are elevated. At the same time, insulin secretion is not activated under normal or low glucose conditions, preventing excessive blood sugar drops (45). Moreover, GLP-1 delays gastric emptying and inhibits glucagon secretion from pancreatic *α*-cells, further contributing to glycemic stabilization. In septic patients, maintaining stable glucose levels is particularly crucial because hypoglycemia can exacerbate cellular damage, intensify oxidative stress, and trigger neuroendocrine stress pathways that disrupt homeostasis. GLP-1 agonists thus help preserve cellular energy balance and reduce inflammatory responses, which correlates with better outcomes in septic patients. This glucose-dependent mechanism makes GLP-1-based therapies safer compared to classical hypoglycemic drugs in critically ill septic patients (46).

### 6.3 Cardioprotective role of GLP-1

Another important aspect is the cardioprotective effect of GLP-1. It is known that sepsis can cause reversible cardiomyopathy and severe microcirculatory disturbances. GLP-1RAs demonstrate multifaceted cardioprotection in sepsis-induced cardiomyopathy through synergistic mechanisms. Direct stimulation of GLP-1R on cardiomyocytes increases cAMP and activates the PI3K/Akt pathway, thereby improving contractility and reducing apoptosis, which preserves left ventricular function and cardiac output (47). Simultaneously, GLP-1 stabilizes the endothelium by decreasing capillary permeability and activating eNOS, leading to increase NO production and improved microcirculation and tissue perfusion. This contributes to blood pressure stabilization, better responses to vasopressors, and optimized oxygen delivery to vital organs (48). Additionally, as mentioned earlier, GLP-1 reduces inflammation (TNF-*α*, IL-6) and oxidative stress, further protecting the myocardium from inflammatory damage.

### 6.4 Neuroprotective role of GLP-1

The neuroprotective effects of GLP-1 receptor agonists are receiving increasing attention in the context of sepsis, as GLP-1 can cross the blood–brain barrier and act directly on the central nervous system. Sepsis often leads to neurological complications such as delirium and sepsis-associated encephalopathy (SAE), which are linked to increased mortality, prolonged intensive care unit stays, and persistent cognitive impairments (49). GLP-1 receptor agonists exert neuroprotective effects primarily by inhibiting microglial activation, the primary effector cells of neuroinflammation, thus reducing the production of proinflammatory cytokines (e.g., TNF-*α*, IL-1β) and alleviating inflammatory damage to brain parenchyma. Furthermore, the activation of intracellular pathways, such as PI3K/Akt and cAMP/PKA by GLP-1RA contributes to decreased oxidative stress, inhibition of neuronal apoptosis, and improved mitochondrial function. Together, these mechanisms help preserve neuronal homeostasis, reduce the risk of delirium and SAE, and potentially improve neurocognitive outcomes in patients with severe sepsis. Moreover, in animal models of neuroinflammation, GLP-1RAs have demonstrated antioxidant, antiapoptotic, and neurotrophic effects; liraglutide prevented postoperative delirium in mice by reducing microglial activation and inflammasome activity (50). Mercado F et al. described the activation of mammalian target of rapamycin (mTOR) as a crucial pathway for neuron protection, and that GLP-1RA affects mTOR activity by reducing caspase-3 and caspase-8 (51). Given that delirium occurs in 30–80% of ICU patients and SAE in over 70% of septic patients, the neuroprotective action of GLP-1 represents an important and underutilized therapeutic potential.

## 7 The dual nature of GLP-1 effects in sepsis

In an overview of GLP-1’s biological functions in sepsis and its emerging therapeutic potential, 27 studies were analyzed, focusing on the organ-protective effects of GLP-1, GLP-1RAs, and DPP-4 inhibitors in *in vivo* models of sepsis. GLP-1 has demonstrated notable immunomodulatory properties by promoting glycolysis over oxidative phosphorylation, thereby reducing the levels of inflammatory mediators, oxidative stress, and organ dysfunction. However, in specific contexts, GLP-1 has also been shown to exhibit cytotoxic effects. Thus, its role in sepsis may represent a “double-edged sword.” During sepsis, nearly all cell types undergo metabolic reprogramming. In the early phase, elevated GLP-1 secretion and receptor upregulation may act as signals of this metabolic shift, redirecting energy production toward glycolysis. While this supports immune cell activation and enhances pathogen clearance, excessive activation may contribute to tissue injury and immunosuppression (52). Brakenridge and colleagues conducted a retrospective analysis of GLP-1 levels in 157 surgical and trauma patients who were admitted to the ICU due to sepsis between January 2015 and September 2016, at the UF Health Trauma Center in Gainesville, Florida. In these patients, serial measurements of GLP-1 and IL-6 were obtained, revealing that elevated GLP-1 concentrations within the first 24 h of sepsis onset were strongly associated with early mortality and the development of chronic critical illness. A similar association was observed in patients who exhibited high GLP-1 levels on day 14 after sepsis onset—they were at increased risk for severe functional impairment or death within the following 6 months. GLP-1 may serve as an indicator of ongoing metabolic stress and unresolved catabolic state, both of which are linked to muscle wasting and the emergence of chronic critical illness after sepsis (53). GLP-1RAs have shown protective effects against pancreatic *β*-cells damage caused by inflammation and SARS-CoV-2 infection. They may help reduce risk factors for severe COVID-19 outcomes-such as obesity, non-alcoholic fatty liver disease, and cardiovascular disease, and are considered potential therapeutic candidates during acute infection for mitigating respiratory damage. Furthermore, they may help prevent chronic lung injuries, including pulmonary fibrosis, in individuals who have had severe COVID-19 (38).

Although numerous experimental studies, primarily in animal models, have demonstrated the beneficial effects of GLP-1 agonists (e.g., liraglutide and exenatide) in sepsis, including reduced inflammation, improved organ function, and prolonged survival, their clinical use in humans is not yet routine and remains under investigation. However, there is growing interest in the potential role of GLP-1RA as adjunctive therapy in the treatment of sepsis, especially in patients with concomitant metabolic disorders.

## 8 Therapeutic implications of GLP-1 agonists in sepsis

We did not find any controlled, randomized study about the therapeutic effects of GLP-1RAs in sepsis as a single treatment. Still, GLP-1RAs could serve as adjunctive therapy in sepsis through several mechanisms: (1) dampening systemic inflammation; (2) improving endothelial function and organ perfusion; (3) optimizing glycemic control without excessive hypoglycemia (54).

These drugs have demonstrated significant multi-organ protective effects in sepsis models by modulating inflammation, oxidative stress, and tissue injury. In the central nervous system, agents like exedin-4 and liraglutide reduce neuroinflammation by inhibiting microglial activation and cytokine production (IL-1β, IL-6, TNF-*α*), while preserving neuronal integrity and preventing cognitive dysfunction associated with sepsis. In the lungs, GLP-1RAs attenuate acute lung injury by maintaining alveolar-capillary barrier function, reducing neutrophil infiltration, and promoting surfactant protein expression, potentially through suppression of NLRP3 inflammasome activity. Hepatic benefits include reduced oxidative stress, inflammation, and improved liver enzyme profiles (ALT, hsCRP), as well as enhanced antioxidant enzyme levels, thereby protecting against acute liver injury. In the kidneys, GLP-1RAs lower urea, creatinine, and pro-inflammatory cytokines via AMPK-dependent pathways, supporting renal function during sepsis-induced acute kidney injury. Cardioprotective actions include the inhibition of TLR4/NF- κB/NLRP3 signaling, reduction of cytokine levels (such as IL-6 and IL- 1β), and prevention of myocardial remodeling and dysfunction. In the vasculature, GLP-1RAs and DPP-4 inhibitors mitigate LPS-induced endothelial permeability, inflammation, and microvascular thrombosis by activating AMPK and CaMKKβ, reducing adhesion molecule expression (VCAM-1, ICAM-1), and improving outcomes in septic shock. These finding support the therapeutic potential of GLP-1RAs as systemic protectant in sepsis (50).

A retrospective cohort study by Alex E. Henney et al. demonstrated that GLP-1RAs are associated with significantly lower risks of pneumonia and severe sepsis in patients with type 2 diabetes compared to dipeptidyl peptidase-4 inhibitors (DPP-4is). Among 331,863 matched GLP-1RAs users, the hazard ratios for pneumonia and severe sepsis were 0.60 (95% CI, 0.58–0.62) and 0.61 (95% CI, 0.59–0.63), respectively, indicating a substantial risk reduction relative to the DPP-1 is cohort (55). A study by Wont et al. demonstrated that GLP-1RAs, such as semaglutide, confer protective effects in polymicrobial sepsis by activating neuronal GLP-1R. In septic mice, semaglutide attenuated sickness behavior, hypothermia, systemic inflammation, and lung injury through the downregulation of pro-inflammatory cytokines (TNF- *α*, IL-1β, IL-6, CXCL1), suppression of neutrophil infiltration, and decreased expression of genes associated with tissue remodeling and inflammation (Mmp9, Timp1, Ly6g). These protective effects were absent in GLP-1R deficient (Glp1r^Wnt1−/−) mice, highlighting the critical role of neuronal GLP-1R signaling in mediating the anti-inflammatory and organ-protective actions of GLP-1RAs in sepsis (56).

Adiponectin, a highly abundant adipokine secreted by adipose tissue, plays a crucial role in regulating metabolism and inflammation due to its anti-diabetic, anti-inflammatory, and vasoprotective properties. In the context of sepsis, experimental studies have demonstrated that adiponectin deficiency exacerbates inflammation, promotes leukocytes and platelet adhesion, and compromises blood–brain barrier integrity, partly through the upregulation of E-selectin. These finding highlight the essential role of adiponectin in modulating microvascular inflammation and maintaining vascular homeostasis during sepsis. Gianoli et al. demonstrated that GLP-1RAs positively regulate adiponectin levels, potentially improving sepsis outcomes by modulating both metabolic homeostasis and inflammatory responses. Mechanistically, GLP-1RAs activated cAMP signaling in adipocytes, resulting in increased adiponectin secretion through the activation of PKA. Exedin-4, a naturally occurring GLP-1RA derived from Gila monster venom, has also been shown to suppress macrophage-driven inflammation by enhancing adiponectin expression. Through this mechanism, GLP-1RAs may reduce endothelial dysfunction and oxidative stress, both of which are key contributors to sepsis pathophysiology (57).

Sepsis-induced muscle weakness is a frequent and debilitating complication that contributes to chronic critical illness. This condition, often classified under ICU-acquired, includes SIM and is characterized by a significant decline in skeletal muscle strength. Atrophy of limb muscles impairs mobility, while involvement of respiratory muscles increases the risk of pulmonary infection and respiratory failure, contributing to poorer outcomes in critically ill patients. GLP-1RAs exhibit multifaceted protective effects in SIM by modulating muscle catabolism, inflammation, oxidative stress, and metabolism. These agents mitigate muscle atrophy by downregulating atrophy-related factors (myostatin, MuRF1, MAFbx) and upregulating myogenic transcription factors (MyoD, MyoG) via PKA/Akt/mTOR signaling. GLP-1RAs attenuate systemic and local inflammation by inhibiting the p38 MAPK/NF-κB pathway, reducing cytokine production, endothelial injury, and microvascular thrombosis. Additionally, they preserve mitochondrial integrity and suppress ROS-induced AMPK-FoxO3 and glucocorticoid pathways, while enhancing antioxidant responses and HSP70 expression. Neuroprotective effects via PI3K/Akt/GSK-3β and IGF-1PI3K-Akt–mTOR signaling further contribute to reduced muscle degradation and improved protein synthesis. By enhancing insulin sensitivity, GLUT4-mediated glucose uptake, and AMPK activity, GLP-1RAs improve skeletal muscle energy metabolism and reduce lipid accumulation in skeletal muscle. The also enhance microvascular perfusion through the activation of PKA-NO and PI3K-Akt-eNOS pathways, promoting the delivery of oxygen and nutrients. Collectively, these mechanisms support the therapeutic potential of GLP-1RAs in preventing and treating SIM (58).

While GLP-1RAs demonstrate promising metabolic and anti-inflammatory effects, their application in treating sepsis remains limited by insufficient clinical evidence, individual variability, and potential side effects. Further research is needed to confirm their efficacy and safety in this context. Given the complexity of sepsis pathophysiology, GLP-1RAs are unlikely to provide comprehensive therapeutic benefits when used in isolation. Instead, they may be more effective as part of multimodal strategy that includes glycemic control, anti-inflammatory interventions, early enteral nutrition, and rehabilitation efforts aimed at preserving muscle mass and function in critically ill patients (59).

## 9 Conclusion

GLP-1 receptor agonists (GLP-1RAs) have emerged as promising therapeutic agents in the management of sepsis and its complications. Their pleiotropic effects, which range from anti-inflammatory, antioxidant, and metabolic regulation to neuroprotective and endothelial-stabilizing properties, are mediated through complex intracellular pathways, including the PI3K/Akt, AMPK, and NF-κB signaling pathways. Preclinical and clinical data support the role of GLP-1RAs in reducing systemic inflammation, improving organ function, and enhancing survival in various sepsis models. However, despite encouraging findings, clinical translation remains limited due to the lack of sufficient human studies, variability in individual responses, and an incomplete understanding of their mechanisms in sepsis. Therefore, further well-designed clinical trials are essential to establish the efficacy, safety, and optimal therapeutic regimens of GLP-1RAs as adjunctive treatment in sepsis.

## Glossary

LMICs: Low- and middle-income countries
HICs: High-income countries
NK cells: Natural killer cells
TNF-α: Tumor necrosis factor-*α*
IL-1: Interleukin-1
IL-6: Interleukin-6
TLR-4: Toll-like receptor-4
LPS: Lipopolysaccharide
NLRP3: NOD-like receptor protein 3
ROS: Reactive Oxygen Species
NO: Nitric oxide
SIM: Sepsis-induced Myopathy
GLP-1R: Glucagon-like peptide-1 receptor
GLP-1: Glucagon-like peptide-1
GLP-1RA: Glucagon-like peptide-1 receptor agonist
PKA: Protein kinase A
PLC: Phospholipase C
ERK1/2: Extracellular signal-regulated kinase 1 and 2
DPP-4: Dipeptidyl peptidase-4
IL-1β: Interleukin-1β
MCP-1: Monocyte Chemoattractant Protein-1
IL-10: Interleukin-10
iNOS: Inducible nitric oxide synthase
eNOS: Endothelial nitric oxide synthase
CRP: C-reactive protein
MDA: Malondialdehyde
8-OHdG: 8-hydroxy-2’deoxyguanosine
ICU: Intensive Care Unit
SOD: Superoxide dismutase
GPx: Glutathione peroxidase
Nrf2: Nuclear factor erythroid 1-related factor 2
SAE: Sepsis-associated encephalopathy
mTOR: Mammalian target of rapamycin
DPP-4is: Dipeptidyl peptidase-4 inhibitors
